# Eye Segmentation Method for Telehealth: Application to the Myasthenia Gravis Physical Examination

**DOI:** 10.3390/s23187744

**Published:** 2023-09-07

**Authors:** Quentin Lesport, Guillaume Joerger, Henry J. Kaminski, Helen Girma, Sienna McNett, Mohammad Abu-Rub, Marc Garbey

**Affiliations:** 1 Department of Surgery, School of Medicine and Health Sciences, George Washington University, Washington, DC 20037, USA; lesport@gwu.edu; 2 Care Constitution Corp., Newark, DE 19702, USA; joerger@orintelligence.com; 3 Department of Neurology & Rehabilitation Medicine, School of Medicine and Health Sciences, George Washington University, Washington, DC 20037, USA; hkaminski@mfa.gwu.edu (H.J.K.); hgirma@mfa.gwu.edu (H.G.); smcnett@gwmail.gwu.edu (S.M.); maburub@mfa.gwu.edu (M.A.-R.); 4 LaSIE, UMR CNRS 7356, Université de la Rochelle, 17000 La Rochelle, France

**Keywords:** telehealth, telemedicine, myasthenia gravis, ptosis, diplopia, deep learning, computer vision, eyes tracking, neurological disease

## Abstract

Due to the precautions put in place during the COVID-19 pandemic, utilization of telemedicine has increased quickly for patient care and clinical trials. Unfortunately, teleconsultation is closer to a video conference than a medical consultation, with the current solutions setting the patient and doctor into an evaluation that relies entirely on a two-dimensional view of each other. We are developing a patented telehealth platform that assists with diagnostic testing of ocular manifestations of myasthenia gravis. We present a hybrid algorithm combining deep learning with computer vision to give quantitative metrics of ptosis and ocular muscle fatigue leading to eyelid droop and diplopia. The method works both on a fixed image and frame by frame of the video in real-time, allowing capture of dynamic muscular weakness during the examination. We then use signal processing and filtering to derive robust metrics of ptosis and l ocular misalignment. In our construction, we have prioritized the robustness of the method versus accuracy obtained in controlled conditions in order to provide a method that can operate in standard telehealth conditions. The approach is general and can be applied to many disorders of ocular motility and ptosis.

## 1. Introduction

Telemedicine (TM) is an emerging tool for monitoring patients with neuromuscular disorders and has significant potential to improve clinical care [1,2], with patients having favorable impressions of telehealth during the COVID-19 pandemic [3,4]. There is great promise in taking advantage of the video environment to provide remote alternatives to physiological testing and disability assessment [2]. Telehealth is particularly well-suited for the management of patients with myasthenia gravis (MG) due to its fluctuating severity and the potential for early detection of significant exacerbations. MG is a chronic, autoimmune neuromuscular disorder that manifests with generalized fatiguing weakness with a propensity to involve the ocular muscles. For this purpose, the Myasthenia Gravis Core Exam (MG-CE) [5] was designed to be conducted via telemedicine. The validated patient reported outcome measures typically used in clinical trials may also be added to the standard TM visit to enhance the rigor of the virtual examination [6]. In this study, we address the first two components of the MG-CE [5], evaluation of ptosis (exercise 1) and diplopia (exercise 2), thus focusing on the tracking eye and eyelid movement.

Today’s standard medical examination of MG relies entirely on the expertise of the medical doctor, who grades each exercise of the protocol by watching the patient. The examiner rates the severity of ptosis by qualitatively judging the position of the eyelid in relationship to the pupil and monitoring for ptosis becoming more severe over the course of the one-minute assessment [7]. The determination of diplopia is entirely dependent on the patient’s report. Further, the exam is dependent entirely on the patient’s interpretation of what is meant by double vision (versus blurred vision) and further complicated by the potential suppression of the false image by central adaptation, and in some situations, monocular blindness, which eliminates the complaint of double vision. The objective of our method is to provide unbiased, automatic quantitative metrics of ptosis and ocular misalignment during the telemedicine visit.

Our long-term goal is to complement the neurological exam with computer algorithms that quantitatively and reliably report information directly to the examiner, along with an error estimate on the metric output. The algorithm should be fast enough to provide feedback in real-time and automatically enter the medical record. A similar approach was used by Liu and colleagues [8], monitoring patients during ocular exercises to bring a computer-aided diagnosis, but with highly controlled data and environment. We aim to use a more versatile approach by extracting data from more generic telehealth footage and requiring as little additional effort from the patient and clinician as possible.

By use of videos, the algorithm should capture the dynamic of ptosis and ocular misalignment over time that is inherent to the neuromuscular fatigue of MG. This feature may not be readily detected by the human examiner, who simply watches the patient perform tasks: the doctor estimates the eye motion at the end of the ptosis exercise versus the initial eye position with a simple scale from 0 to 3. For the diplopia exercise, the doctor enters in the report if the patient experienced double vision or not because of the great difficulty in judging ocular eye misalignment during a telemedicine visit.

It is understood that the medical doctor is the final judge of the diagnostic. Our method is a supporting tool, as are AI generated image annotations in radiography [9], and it is not intended to replace the medical doctor’s diagnostic skill. Further, our approach does not supplement the sophisticated technology used to study ocular motility for over five decades [10].

Symptoms of double vision and ptosis are expected in essentially all patients with MG, and the evaluation of lid position and ocular motility is a key aspect of the diagnostic examination and ongoing assessment of patients, with ocular myasthenia being the most common first form of the disease, before progressing to the generalized form [7]. In many neurological diseases, including dementias, multiple sclerosis, stroke, and cranial nerve palsies, eye movement examination is important in diagnosis. We expect that our algorithm will also be useful in the diagnosis and monitoring of many neurological disorders via telehealth [11,12,13]. The technology may also be adapted for assessment in the in-person setting as a means to objectively quantitate the ocular motility examination in a simple fashion.

The manuscript is organized as follows: First, we provide background on the development of our study. Second, we describe in detail our method and algorithms and place into context of previous studies to illustrate the novelty of our approach. Then we report the results followed by discussion. Finally, we summarize our finding in the conclusion and provide some recommendations on follow-up investigations.

## 2. Background

**Figure 1:**
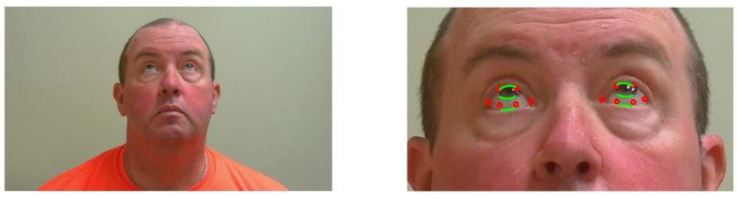
Subject looking up for evaluation of ptosis with identification of key landmarks. The red dots are the landmarks obtained with the machine learning method. The green lines correspond to the detected position of upper and lower eyelids and the iris boundary.

The NIH Rare Disease Clinical Research Network dedicated to myasthenia gravis (MGNet) initiated an evaluation of examinations performed by telemedicine. The study recorded the evaluations, including the MG Core Exam, to assess reproducibility and exam performance. We took advantage of the Zoom recordings performed at George Washington University to evaluate our technology. We used two videos of each subject performed on different days for quantitative assessment of the severity of ptosis and diplopia for patients with a confirmed diagnosis of MG. The patients were provided instructions regarding their position in relationship to their cameras and levels of illumination, as well as to follow the examining neurologist’s instructions during the examinations. In exercise 1, the patient is asked to hold their gaze upwards for 61 s (see Figure 1). The goal is to assess the severity of ptosis (uncontrolled closing of eyelid), if any, during the exercise [14] and rating severity:

0: No visible ptosis within 45 s

1: Visible ptosis within 11–45 s

2: visible ptosis within 10 s

3: Immediate ptosis

However, another grading system was proposed for the MG-CE using the following ratings:

0: No ptosis

1: Mild, eyelid above pupil

2: Moderate, eyelid at pupil

3: Severe, eyelid below pupil

**Figure 2:**
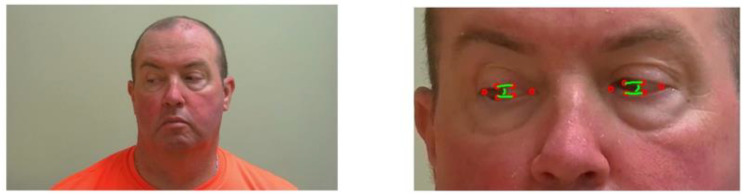
Subject looking eccentrically in exercise 2 to evaluate for development of diplopia.

In exercises 2, the patient must hold their gaze right or left for 61 s—see Figure 2. The goal is to assess for double vision, and when it appears. Severity rating ranges from 0 to 3:

0: No diplopia with 61 s sustained gaze

1: Diplopia with 11–60 s sustained gaze

2: Diplopia within 1–10 s but not immediately

3: Immediate diplopia with primary or lateral gaze

Our goal was to take accurate and robust measurements of the eye anatomy in real-time during the exercises, and to automatically grade ptosis and ocular misalignment. The algorithm should reconstruct the eye geometry of the patient from the video and the position of the pupil inside that geometric domain. The difficulty is to precisely recover these geometric elements from a video of the patient where the eye dimension in pixels is at best about 1/10 of the overall image dimension. Most of the studies of oculometry assume that the image is centered on the eye and that it occupies most of the image. Alternatively, eye trackers do not rely on a standard camera using the visual spectrum but rather use infrared in order to isolate clearly the pupil as a feature in a corneal reflection image [15,16,17].

Localization of eye position can take advantage of deep learning methods but requires large, annotated data sets for training [18,19]. As described later, we use an existing open-source library to take advantage of such information. From a model of eye detection, we can focus the search for pupil and iris location in the region of interest [20]. Among the popular techniques to detect the iris location [21] are the circular Hough transform [22,23] and the Daughman’s algorithm method [24].

We found in our telehealth application, using a standard camera operating in the visual spectrum, that these methods have a robustness issue due to their insensitivity to low resolution of the eyes’ Region Of Interest (ROI), poor control on illumination of the subject, and specific eye geometry consequent to ptosis. Next, we will present our hybrid method, combining an existing deep learning library for face tracking and a local computer vision method to build ptosis and diplopia metrics.

## 3. Method

### 3.1. Dataset

We used twelve videos acquired by Zoom during the ADAPT study telehealth sessions of six patients with MG. Each subject had telemedicine evaluations within 48 h of each other and participated in a set of standardized outcome measures including the MGNet Core Exam [5]. Telehealth sessions were organized as Zoom meetings by a board-certified neurologist with subspecialty training in neuromuscular disease in the clinic, providing the assessments of all patients at their homes. In practice, these Zoom sessions were limited in video quality to a relatively low resolution in order to accommodate the available internet bandwidth and because they were recorded on the doctor side during streaming. We extracted fixed images at various steps of the exercise to test our algorithm, as well as on video clips of about 60 s each for each exercise 1 and 2, described above. The number of pixels per frame was as low as 450 × 800 at a rate of 30 Frames Per Second (FPS). We also included half a dozen videos of healthy subjects acquired in similar conditions to the ADAPT patients in order to have a base line on eye motion during both eye tracking exercises.

We aimed to achieve 2-pixel accuracy of anatomic markers, allowing us to compute ptosis and eye alignment. This was chosen for the following reasons: (1) When one looks for an interface location, two pixels is about the best accuracy one may achieve since it is projected on a discrete grid of pixels; (2) this metric is independent of the resolution of the image. As a matter of fact, there is no control of the dimension of eyes expressed in pixels in a video frame during the telemedicine session, since the assessment is posteriori on existing videos; (3) in addition, high definition camera footage with a patient far away from the camera will have lower resolution of ocular anatomy than a standard Zoom recording with a patient close to the camera, as the eye dimension is expressed in pixels.

The distance of the patient to the camera and illumination of the subject leads to variability of the evaluations. These conditions are inherent limitations of the telehealth standard to accommodate patients’ equipment and home environment.

We used our new telehealth platform, which includes a Lumens B30U PTZ camera (Lumens Digital Optics Inc., Hsinchu, Taiwan) with a resolution of 1080 × 1920 at 30 FPS and is connected to a Dell Optiplex 3080 small form factor computer (Intel processor i5-10500 t, 2.3 GHz, 8 Gb Ram), for processing video analysis. This system, tested initially on healthy subjects, was used on one patient, following the MG-CE protocol. Through this process, we have acquired a data set that is large enough to evaluate the robustness and quality of the algorithms. Error rates depending on resolution and other human factors are compared in the Section 4. Next, we describe the algorithm construction.

Full session videos were divided into short clips of each exercise using an editing software. The clips were then processed one at a time. For every applicable exercise, the video went through the Machine Learning algorithm, saving landmark positions of the face using [15] in Python. Once the landmarks were saved, they were exported to a Matlab script where the rest of the processing using conventional computer vision methods coded in Matlab was performed. All processes were executed automatically on a laptop. We can also use the Matlab compiler to accelerate processing and protect the software from alterations.

### 3.2. Face and Eyes Detection

The first step was to detect the face in the image. Previous investigations have developed face tracking algorithms and compared methods for facial detection [25,26]. Among the most widely used algorithms and the one we chose to use was OpenCV’s implementation of the Haar Cascade algorithm [27], based on the detector of R. Lienhart [28], which is a fast method and, overall, the most reliable for real-time detection.

**Figure 3:**
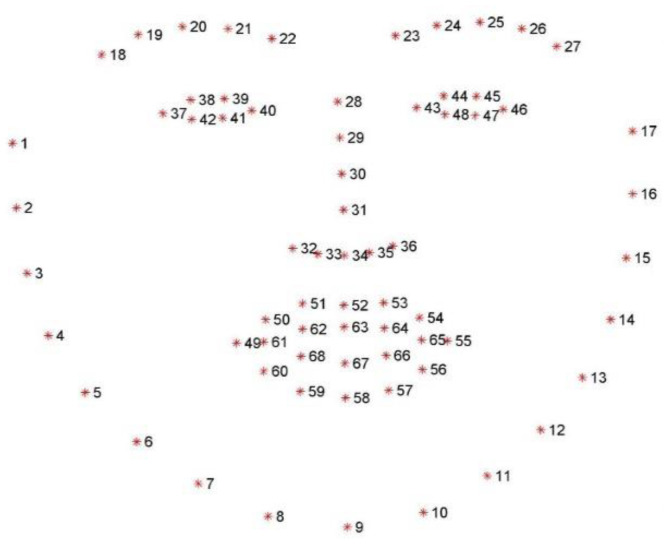
Dlib Facial Landmarks [15].

**Figure 4:**
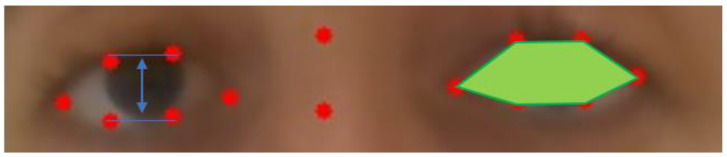
Eye Opening distance (blue arrow) and eye area (in green), obtained from the machine learning landmarks (red dots).

Once the bounding box of the face is detected, key facial landmarks were required to monitor the patient’s facial features, which would be the foundation of our segmentation and analysis to evaluate ptosis and ocular misalignment. For facial alignment, many methods exist. Some of these image-based techniques were reviewed by Johnston and Chazal [29]. One of the most time-efficient for real-time application is based on the shape regression approach [30]. We used DLib’s implementation of the regression tree technique from V. Kazemi and J. Sullivan [31], which was trained on the 300 W dataset [32] fitting a 68 points landmark to the face (Figure 3 and Figure 4). The ROI for each eye is the polygon formed by points 37 to 42 for the right eye, and 43 to 48 for the left eye, in reference to the model in Figure 3.

We present below the postprocessing of each video frame, concentrating on the ROI specific to each anatomic landmark (upper lid, lowerlid, and part of the iris boundary). While in principle, processing of the video instead of serial images one by one would be advantageous, blinking and rapid eye movements interferes with the process and make a robust solution more difficult to build.

#### 3.2.1. Computing the Ptosis Metric

First, we processed the time window of the video clip when the patient is focusing eye gaze upwards.

**Figure 5:**
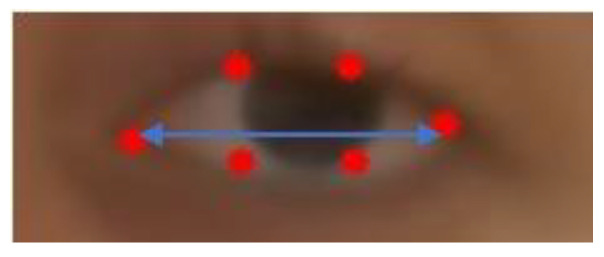
Eye length measurement.

The ROI construction for each eye may give a first approximation of ptosis for exercise 1 of the MG-CE as follows: We used eyelid distance and eye area, as shown in Figure 4. The eyelid distance (ED) approximation is the average distance between points of the upper eyelid (segment 38–39 for the right eye and 44–45 for left eye) and lower eyelids (segment 42–41 for the right eye and 48–47 for left eye), as determined by the landmarks in Figure 3. Eye area is the area contained in the outline of the eye determined by the landmark (Figure 4). We normalize these measurements by the eye length (EL), as the horizontal distance between the corners of each eye (Figure 5).

**Figure 6:**
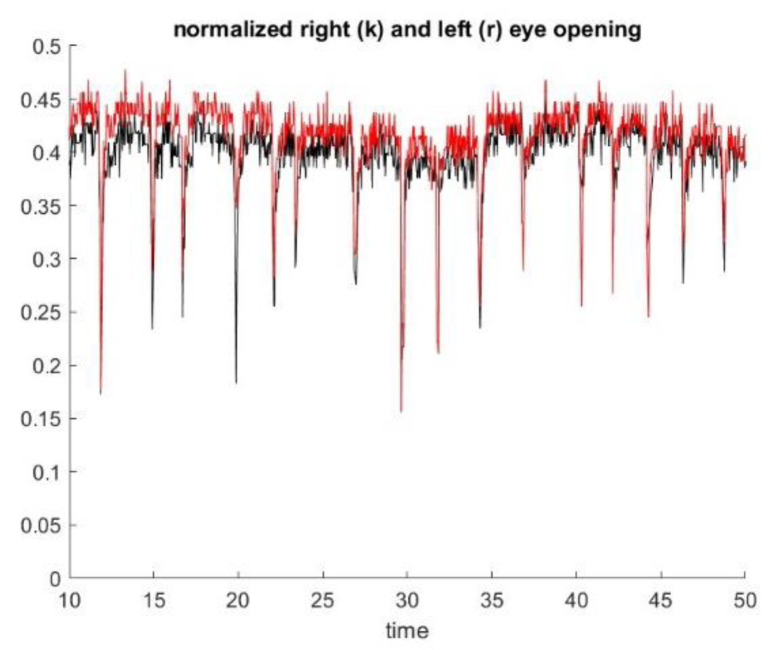
Blinking Identification: The downward spikes of the graph are perfectly synchronized and correspond to blinks.

As a byproduct, we also identify the blink rate, as shown in Figure 6.

**Figure 7:**
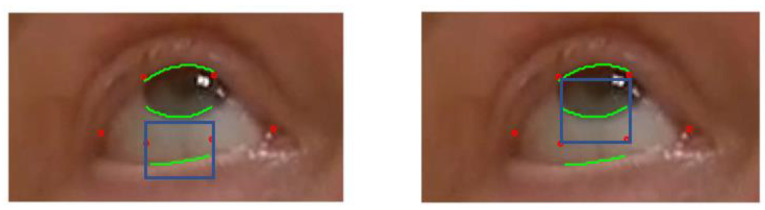
Local Rectangle on left to search for the correct position of the lower lid and on right to draw the interface between the iris and sclera below. The red dots are obtained with the machine learning method. The blue squares are the regions used to search for the green landmarks: the iris boundary and the upper and lower eyelids.

The eye lid location provided by the deep learning algorithm may not be accurate, as shown in Figure 7. For example, here the lower landmarks (41) and (42) are quite far off the contour of the eye, and landmarks (37) and (40) are not precisely localized at the corners of the eye. The accuracy of this library varies depending on the characteristic of the subject, such as iris color, contrast with sclera, skin color, etc. The accuracy is also dependent on the frame of the video clip and effect of lightning or variations of head position. We also found that the hexagon of the model identified by the deep learning algorithm may degenerate to a pentagon when a corner point overlaps another point of the hexagon. In some extreme cases, we found the ROIs at the wrong location; for example, the algorithm confused the nares with the eye location. Such an error was relatively easy to detect but improving the accuracy of the deep learning library for a patient performing an eccentric gaze position, such as in exercise 1 and 2, would require re-training the algorithm with a model having a larger number of landmarks concentrating on the ROI. This is feasible but would require a large traing set. MG is a rare disease, and no large data sets suitable for training a deep learning techniques exist at present. Further, we have no way to predict the size of data set necessary to effectively train the algorithm.

Many eye detection methods have been developed in the field of ocular motility research, but they rely on images taken in controlled environments with specific infrared lights allowing for maximal contrast to define the eye and orbital anatomy, and are focused exclusively on the eye.

The essential feature of our approach is to start from the ROI, i.e the polygons provided by deep learning that delineate roughly the eye “contour”. This result is relatively robust with a standard video but is not very accurate overall. Therefore, we zoomed in on spatial subdomains of the ROI in a divide and conqueer manner to segment each interface individually using standard computer vision algorithms. Special features are the upper lid/lower lids and bottom of the iris boundary for ptosis and the visible side of the iris boundary for diplopia. We have separated the upper lid, lower lid, and the iris boundaries as we are looking for a single interface in each rectangular box, as in Figure 7. In principle, these interface boundaries should cross the rectangle horizontally for lid position and vertically for ocular misalignment. One must check with an algorithm that the interface partitions the rectangle into two connex sub domains. The segmentation algorithm may shrink the rectangle to a smaller dimension as much as necessary to separate each anatomic feature.

To be more specific, we first describe the local search method to position the lower lid and the lower boundary of the iris during ptosis exercise 1. The description below is set for the right eye, with the processing of left eye being analagous. As shown in Figure 7, to improve the lower lid positioning, we draw a small rectangle including the landmark points (42) (41) and look for the interface between the sclera and the skin of the lower lid. Similarly, we draw a rectangle that contains (38), (39), (40), and (41) and identify the interface of the iris and sclera.

The interface found by the computer vision algorithm would be acceptable only if it is a smooth curve (H1) that crosses the rectangle horizontally (H2). For the iris bottom, we expect the curve to be convex (H3).

As the computer vision is concentrated in a rectangle of interest that contains the interface we are looking for, the problem is simpler to solve, and the solution can be found accurately provided that the two subdomains separated by the interface have clear distinct features. To that purpose, we first enhance the contrast of the image in that rectangle before further processing. Second, we used several simple techniques, such as kmeans, restricting ourselves to two clusters, or open snake, which maximizes the gradient of the image along a curve. Those numerical techniques come with numerical indicators to show how well two regions are clearly separated in a rectangular box. For example, with the kmean algorithm, we center the two clusters clearly separated, and each cluster should be a connex set (H4). For the open snake method, we check on the smoothness of the curve and the gradient value across that curve.

If the computer vision algorithm fails to find an interface that satisfies all our hypotheses (H1 to H4), we either reran the k-means algorithm, changing the seed, or shrink the size of the rectangle until convergence to an acceptable solution. If the computer vision algorithm fails to find a smaller rectangle that fits the search, we cannot conclude on the lower lid and upper lid position and must skip that image frame from our reconstrution of the ptosis/diplopia metrics we are building. This may happen if there is a lack of contrast due to poor illumination or a diffuse image due to motion.

The kmean algorithm and snake are some of the simplest algorithms used to separate the two subdomains of the rectangle and draw an interface that satisfies the criteria (H1) to (H4). We used both algorithms simultaneously to determine concurrence of each algorithm. As shown in the Section 4, this solution is robust and has satisfactory accuracy. There is no difficulty in using a more sophisticated level set or graph cut technique, other than the additional cpu requirement.

In the example seen in Figure 7, the model provides the correct location of the upper lid, and the contrast between the iris and the skin above is clear.

Overall, our hybrid algorithm combines deep learning with local computer vision output metrics, such as the distance between the lower lid and the bottom of the iris as well as the lower lid and the upper lid. The first distance is useful to determine that the patient is performing the exercise correctly, while the second distance provides an assessment of ptosis. It is straightforward to assess the diameter of the iris as the patient is looking straight and the pupil should be at the center of the iris circle.

#### 3.2.2. Computing the Diplopia Metric

**Figure 8:**
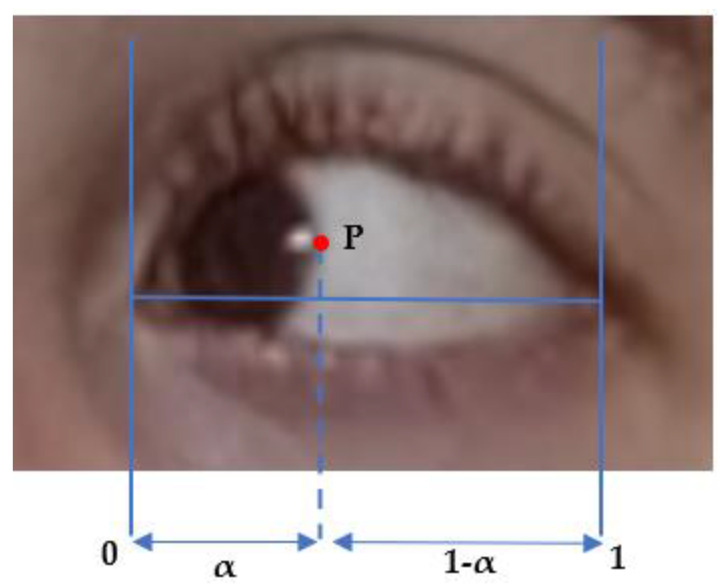
Barycentric Coordinate (α) used in Diplopia Assessment.

As illustrated in Figure 2, we use a similar method to identify the upper lid and lower lid position. The only novelty here is to identify the correct side boundary of the iris as the patient is looking left or right, using a computer vision algorithm in a small horizontal box that starts from the corner of the eye landmark (37) or (40) and goes to the landmarks of the upper lid and lower lid on the opposite side, i.e., (39) and (41) or (38) and (42). The same algorithm is applied to the right eye, as described above, and the left eye, except that the inteface is now crossing the rectangle of interest vertically. We compute the barycentric coordinate denoted α and the horizontal distance of the point P that is the visible lateral point of the iris boundary to the corner of the eye, relative to eye length, as shown in Figure 2 and Figure 8. We denote these as αleft and αright the measurement α for the left and right eyes, respectively. The distance from the face of the patient to the camera is much larger than the dimension of the eye and makes the barycentric coordinate quasi-invariant to the small motion of the patient head during the exercise.

In principle, Pleft and Pright should be of the same order, as the subject is looking straight at the camera; αleft and αright should also be strongly corelated, as the subjects direct their gaze horizontally. As fatigue occurs, the difference between αleft and αright should change with time and correspond to the misalignment of both eyes. We assume that diplopia occurs when the difference between αleft–αright deviates significantly from its initial value at the beginning of the exercise.

#### 3.2.3. Eye Gaze and Reconstruction of Ptosis and Diplopia Metrics in Time

Thus far, we have described a hybrid algorithm that we used for each frame of the video clips during Exercise 1 and 2. As before, we used a simple clustering algorithm for the ROI for each eye to reconstruct the scleral area and detect the time window for each exercise: the sclera should be one side left or right of the iris in Exercise 2 and one side below the iris in Exercise 1. Since we know, a priori, that each exercise lasts one minute, we do not need a very accurate method to reconstruct when the exercise starts or ends. For verification purposes, the result of left eye gaze and right eye gaze should be consistent.

We cannot guarantee that the computer vision algorithm converges for each frame.

We are required to check for:

- Stability: the patient should keep their head in approximately the same position
- Lightning effects: the k-means algorithm shows non-convex clusters in the rectangle of interest when reflecting light affects the iris detection, for example.
- Instability of the deep learning algorithm output: when the landmarks of the ROI change in time independently of the head position.
- Exception of quick eye movements due to blinking or reflex that should not enter the ptosis or diplopia assessment.

As our method eliminates all the frames that do not pass these tests, we generate a time series of measures for ptosis and diplopia during each one-minute exercise that is not continuous in time. Rather, we use linear interpolation in time to fill the holes and provide that the time gaps are small enough, i.e., a fraction of a second. All time gaps that are greater than a second are identified in the time series and may correspond to marker fatigue during the evaluation.

To obtain the dynamic of the ptosis and diplopia measure that is not part of the standard core exam, and to present some interest for neuromuscular fatigue, we further postprocess the signal with a special high order filter, as in [13], that can take advantage of Fourier technique for nonperiodic time series.

## 4. Results

To construct the validation of our method, we visually compare the result of our hybrid segmentation algorithm to a ground true result obtained on fixed images. In order to obtain a representative data set, we extract an image every two seconds from the videos of patients. We used six videos of the ADAPT series with the first visit of six patients. The subjects were three women, three men—one African American/Black, one Asian, and three White, with one person who identified as Hispanic.

We extracted one image every 2 s of the video clip for Exercise 1, assessing ptosis, and the two video clips corresponding to Exercise 2, assessing ocular misalignment. We conducted the same analysis with the patient video that was recorded with the Inteleclinic system equipped with a high-definition camera.

Each exercise lasts about one minute, and we obtained about 540 images from the ADAPT series and 90 from the Inteleclinic. The validation of the image segmentation was performed for each eye, which doubles the amount of time.

For Exercise 1, we check three landmarks positions: the points on the upper lid, iris bottom, and lower lid situated on the vertical line that crosses the center of the ROI. For exercise 2, we identify the position of the iris boundary that is opposite to the direction of gaze. If the patient looks to their left, we determine the position of the iris boundary that is furthest to the right.

To facilitate the verification, our code automatically generates these images with an overlay of the grid of spatial steps at two pixels. This rule is used vertically for exercise 1 and horizontally for exercise 2.

**Figure 9:**
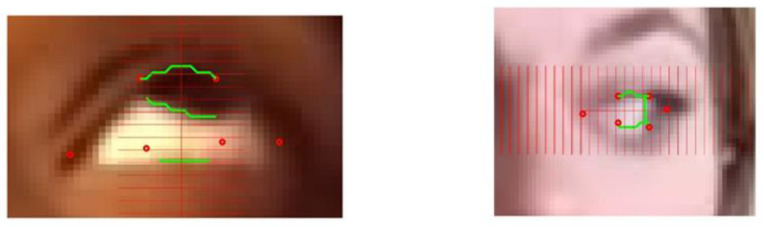
Visual verification on zoomed image of eyes using a 2-pixel rule for Exercise 1 on left, Exercise 2 on right. Green lines are the detected positions of upper and lower eyelids and iris boundary. The red dots correspond to the landmarks detected with the machine learning method.

We consider that the segmentation is correct to assess ptosis and ocular misalignment when the localization of the landmarks is correct within two pixels. It is often difficult to judge the results based on our own inspection, as demonstrated in the image of Figure 9. We used independent visual verifications by two reviewers to make our determinations.

Not all images are resolved by our hybrid algorithm. It is critical to have an adequate time frame in the video to reconstruct the dynamic of ptosis and ocular misalignment. First, we eliminated all the images from the data set in which the Deep Learning library failed to correctly localize the eyes. This can be easily detected in a video, since the library operates on each frame individually and may jump from one position to a completely different one while the patient stays still. For example, for one of the patients, the deep learning algorithms confused each of the nostrils with the eyes.

The MG-CE videos had low resolution, especially when the displays of the patient and the medical doctor were side by side, or have poor contrast, focus, or lighting. Therefore, it is impressive that, on average, we were able to use 74% of the data set for further processing with our hybrid algorithm.

Our algorithm cannot precisely find the landmark of interest when the deep learning library gives an ROI that is significantly off the target. The bias of the deep learning algorithm was particularly significant during exercise 1, in which the eyes are wide open, and the scleral area is decentered below the iris. The lower points of the polygon that mark the ROI are often far inside the white scleral area above the lower lid. The end points of the hexagon in the horizontal direction may become misaligned with the iris too far off the rectangular area of local search we wish to identify.

We automatically eliminated 44% of the images of the video clips of the ADAPT series and 10% of the Inteleclinic series for exercise 1. The Inteleclinic result was acquired in better lightning conditions and with a higher resolution than the ADAPT videos.

We consider that the segmentation is correct if the three landmarks of the ptosis exercises, as defined earlier, are within two pixels of the ground true result obtained manually.

For exercise 1 with the ADAPT series, we obtained a success rate for identification of 73% for the lower lid, 89% for the bottom of the iris, and 78% for the upper lid. For exercise 1 and for the Inteleclinic series of images, we obtained a success rate of 77%, 100%, and 77%, respectively.

For exercise 2, the quality of the acquisition was somewhat better. We eliminated 18% of the image ROIs for the ADAPT series and 13% for the Inteleclinic series.

The localization of the iris boundary, used to check ocular misalignment, was better, with a success rate of 95%. The eyelids are less open than in exercise 1 and are held in the primary position. The upper and lower lid landmarks were obtained with a success rate of 73% and 86%, respectively.

Overall, the success rates between the ADAPT series, provided by standard Zoom call, and the Inteleclinic system result, which uses an HD camera, were similar. Under the same conditions of distance between the patient and the camera, as well as the 2-pixel accuracy, Inteleclinic may provide a submillimeter accuracy of eye motion that is remarkable and much better than the ADAPT series result. However, the Inteleclinic is more sensitive to the head motion of the subject.

We now present summary results of the ptosis and diplopia assesments of MG subjects, which were the goal of the eye and lid motion algorithm development. 

As illustrated in Figure 3, we can determine, from the polygon obtained by the deep learning algorithm, a first approximation of ptosis severity by computing the area of the eye that is exposed to the view, as well as the vertical dimension of the eye. As a byproduct of this metric, we can identify blinking (see Figure 6). We appreciated that left and right eye blinking occur simultaneously, as is expected. Not every subject with MG blinks during the exercise.

**Figure 10:**
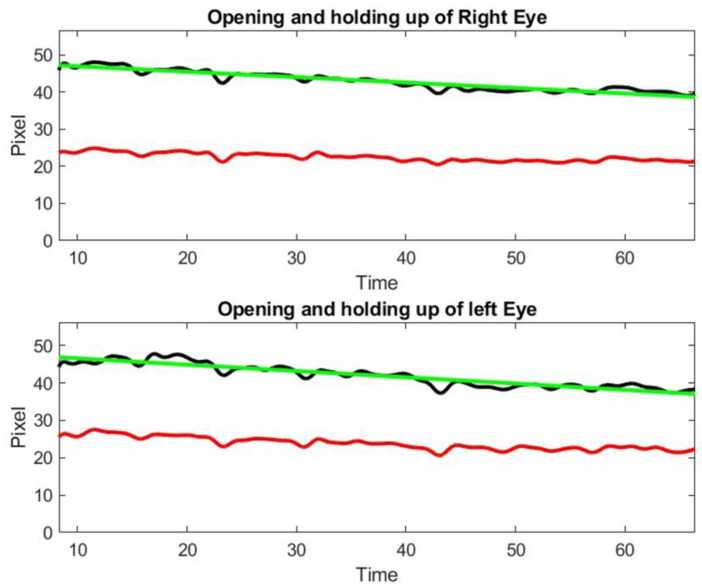
Linear least square fitting coefficient of the upper lid drop from right and left eye, respectively = [−0.15, −0.17]. The green line shows a least square approximation of the distance between the lower lid and upper lids of the patient. The red curve shows the distance between the lower point of the iris and the lower lid below. This second curve is used to check that the patient performs the exercise correctly.

The time dependent measure of diplopia or ptosis obtained by our algorithm contains noise. We can improve the accuracy of the measures by ignoring the eyes with identified detection outliers, provided that the time gaps corresponding to these outliers are small. To recover the signal without losing accuracy, we use the same high order filtering technique that we used in a previous paper to analyze thermal imagery signal [13]. The Inteleclinic data set is working well, as shown in Figure 10.

We observe a 15% decay in lid opening that is very difficult to appreciate by human inspection of video clip or during the in person medical doctor examination. As a matter of fact, this low shift of the upper lid is slow and almost unnoticeable during a 60 s observation. This patient was considered as asymptomatic during the neurologist examination, but the lid droop has been identified by our method. Control subject patients, even the elderly, did not exhibit lid drop of a similar degree during the ptosis exercise.

**Figure 11:**
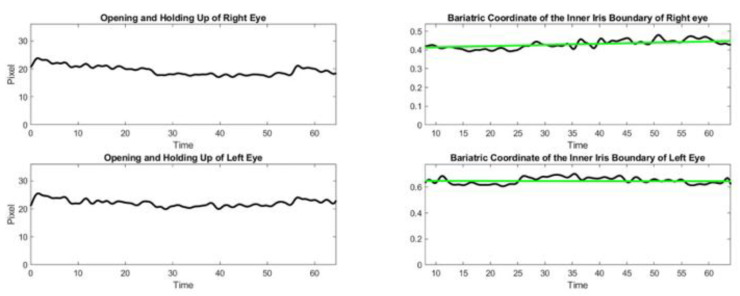
Evolution of the barycentric coordinates for each eye during exercise 2. The green line is the least square fitting of the barycentric coordinates evolution. It is perfectly horizontal which means no weakness in eye motion was shown during the exercise.

During exercise 2 with the patient of the inteleclinic data set, we obtained no eye misalignment (see Figure 11), but the eye opening was about half of its value during the first ptosis exercise, and the eye opening does not stay perfectly constant. We observe, on the Inteleclinic video, that the eye gaze direction to the left and to the right is so extreme that one of the pupils might be covered, in part, by the skin at the corner of the eyes.

**Figure 12:**
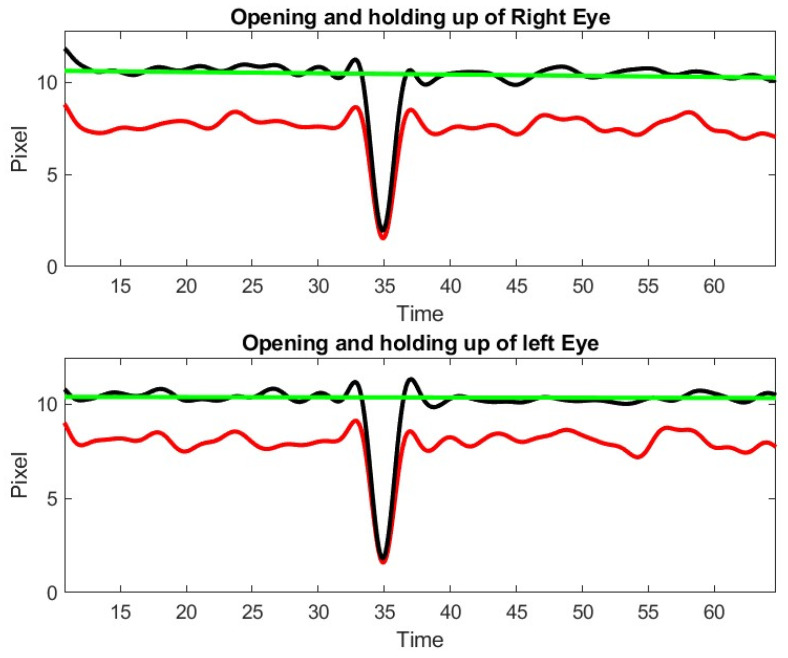
Example of the assessment of one the ADAPT patient series. Note the patient closes his eyes at 35 s.

The results of ptosis and diplopia assessments from the ADAPT video were less satisfactory but still allowed an assessment of lid droop and ocular misalignment, though with less accuracy. Figure 12 shows a representative example of the limits of our approach, when the gap of information between two time points cannot be recovered. It should be appreciated that the eye opening was of the order of 10 pixels as opposed to about 45 pixels in the Inteleclinic data set. In this situation, the subject was not close enough to the camera, which compromised the resolution significantly. However, a posteriori review identified the gap identified by our algorithm and it corresponded to a short period of time when the patient stopped looking up to rest from looking straight. It should be appreciated that holding an upward gaze for one-minute is a strenuous atypical activity for anyone.

## 5. Discussion

Due to the precautions caused by the COVID-19 pandemic, there has been a rapid increase in the utilization of telemedicine in patient care and clinical trials. The move to video evaluations offers the opportunity to objectify and quantify physical examination, which presently relies on the subjective assessment of an examiner with varied levels of experience and often limited time to perform a thorough examination. Physicians still remain reticent to incorporate telemedicine into their clinical habits, in particular in areas that require a physical examination (neuromuscular diseases, movement disorders) compared to areas that are primarily symptom-focused (headache). Telemedicine, on the other hand, has numerous features that can provide an enhanced assessment of muscle weaknesses, deeper patient monitoring and education, reduced burden and cost of in-person clinic visits, and increased patient access to care. The potential for clinical trials to establish rigorous, reproducible examinations at home provides similar benefits for research subjects.

MG is an autoimmune, neuromuscular disease. Outcome measures are established for MG trials, but these are considered suboptimal [33]. The MG CE, in particular for ocular muscle weakness, has been standardized and is well defined [5]. Because of the benefit for frequent monitoring for MG patients, reliable and quantitative teleconsultation would be highly valuable for patient care. However, the grading of ptosis and diplopia relies on repetitive and tedious examinations that the examiner must perform. The dynamic component of upper eyelid dropping can be overlooked during the typical examination by non-experts. Diagnosis of diplopia in these telehealth sessions relies on subjective patient feedback. Overall, the physical examination relies heavily on qualitative, experienced judgment, rather than on unbiased, rigorous, quantitative metrics.

Our goal is to move from 2D teleconsultation and its limitations to a multi-dimension consultation. The system presented in this paper addresses that need by introducing modern image processing techniques that are quick and robust in the process of recovering quantitative metrics that should be independent of the examiner. The diagnosis and treatment decisions remain the responsibility of the medical doctor, who has the knowledge, and not our algorithm output.

One of the difficulties of standard telehealth sessions is the poor quality of video. The resolution may be severely limited by the bandwidth of the network at the patient location. Zoom video footage is also limited by the poor compression/decompression methods used, which could cause artifacts with respect to the original raw video recording acquired by the patient’s camera. Eventually the patient evaluation would benefit from software used on their computers or tablets in order to directly compute all exam metrics with the resolution of the native image before any compression occurs. These metrics, which are just a few numbers, can be sent on a network directly to the physician without bandwidth limitations In our study, the quality of the video was adequate to allow the medical doctor assess ptosis and diplopia as specified by the protocol, but not ideal for image processing, especially because the videos were recorded on the doctor side rather than recording the raw video footages on the patient side. Light conditions and positioning of the patient in front of the camera was often poorly controlled when patients are at home with their personal computer or tablet. These factors are all of crucial importance to optimize numerical algorithm and image processing that are robust and transparent on the level of accuracy they provide. Eye tracking is highly sensitive to patient motion, poor resolution, and eyelid droop with gaze directed eccentrically. However, use of standard telemedicine video will be necessary, despite limitations, to support scalability and adoption of our approach for broad use by physicians and patients.

As we digitalize the exercise output for assessing ptosis, we must rigorously define the metric. We assessed instantaneous measurements as well as time dependent ones. From the dynamic perspective, we could identify patients who show steady upper eyelid position from those who start well and develop progressive eyelid weakness. We also separate global measurement related to the overall eye opening from measurement that computes the distance from the pupil to the upper lid. This last metric is clinically significant for the patient when the droop covers the pupil and impairs vision. A decision on how these metrics should classify ptosis severity remains the physician’s decision.

Similarly, we assumed that diplopia could be measured by the “misalignment” of the left and right pupil during exercise 2. Vision involves not only the alignment of the ocular axes but also the brain’s ability to compensate for such the misalignment and eliminate the complaint of double vision.

Both measurement of ptosis and diplopia were quite sensitive to the resolution of the video. In Zoom recorded telehealth sessions, the distance from the pupil to the upper lid is of the order of ten pixels. A two pixels error on the landmark positions may still provide a relative error of about 20% on the ptosis metric. The deep learning algorithm introduces even larger errors on the landmark points of the ROI polygon. However, with the HD camera we tested, and the processing being conducted on raw footage rather than on streamed recorded footage, this relative error is divided by two. Our analytical approach has also been able to provide recommendations on how to improve the MG ocular exam. For example, to ensure the reproducibility and quality of the result, our algorithm can provide feedback in real-time to the medical doctor on how many pixels are available to track the eyes and therefore give direction to the patient to position closer and better with respect to the camera on their end. Similarly, exercise 2 may benefit from reduced extreme eccentric gaze, which compromises definition of iris boundary, when covered by the overlying eyelid. This would allow for a more appropriate situation to assess double vision properly.

The general concept of our hybrid approach, starting from deep learning to obtain the ROI, and zooming into specific regions of the eye using computer vision, might be applied to assess eye movements in other neurological diseases [34]. However, this would require significant additional analysis to adapt the method to a higher requirement on data acquisition, for example in application of assessment of saccades or smooth pursuit.

## 6. Conclusions

We have presented a hybrid algorithm to estimate the eye and lid movements of patients with myasthenia gravis using the established MG core examination. Our hybrid algorithm starts from a standard machine learning algorithm in order to obtain a coarse estimate of the eye contour with a hexagon. This off the shelf deep learning library is not trained for ptosis and diplopia evaluation that changes dramatically with eye position, but it provides a coarse estimate on eye “contour” in a robust way. We then use a divide and conquer technique to define smaller rectangles that separate each anatomic marker of interest, such as upper lid, lower lid, and part of iris boundary, needed to estimate the lid and eye position. Because the problem of segmentation becomes much simpler in each local rectangle, we can use a standard image segmentation technique and check that the segmentation passes several tests to eliminate artifacts due to head motion, poor lightning condition, and lack of image resolution in a frame-by-frame manner.

We assessed a large, representative data set of image frames that our algorithm delivered with a two-pixel accuracy in about 80% of cases, which is adequate to filter outliers and compute ptosis and eye alignment metrics. As opposed to the standard MG core examination, our method provides the dynamic evolution of ptosis and ocular alignment during the standard 60 s exercise of the standard MG core examination. This information is usually not accessible quantitatively to the neurologist during a standard telemedicine visit, and has proven to be more sensitive than the neurologist assessment on several occasion.

It should be noted that our method highlights a few open clinical questions: how do we make use of the time dynamic evolution of ptosis and diplopia during the exercises? Is it meaningful to capture the fatigue effect or is overall observation by the examiner adequate?

Our methods have limitations, and there is still much room for improvement. Development of a model of the eye geometry with iris and pupil geometric markers that extend the model of Figure 3 in greater detail, including upper lid droop, is necessary. Applying deep learning technology to this model would be quite feasible. This is certainly a worthy effort, but it would require hundreds of patient videos with correct annotation to train the algorithm [19]. However, given the expected expansion of telemedicine with ongoing limitation of a patient’s technology and internet access, this is an important endeavor. Further, as we have seen, deep learning technology may have spectacular robustness that were shown in annotated videos but may not ensure accuracy.

We currently are developing a high-performance telehealth platform [35] that can be conveniently distributed at multiple medical facilities in order to build the large, annotated quality data set that may help advance our understating of MG.

## 7. Patents

A smart Cyber Infrastructure to enhance usability and quality of telehealth consultation, M. Garbey, G. Joerger, provisional 63305420 filed by GWU, January 2022 and as a PCT/US23/61783, January 2023.

## Data Availability

Study videos are recorded on Zoom software. The video recordings are downloaded and stored in secure-HIPAA compliant dropbox and GW MFA servers. Study data is entered into REDCap, a secure web-based data collection system. The REDCap system complies with all applicable guidelines to ensure patient confidentiality, data integrity, and reliability.
